# Synthesis of Two Tetrasaccharide Pentenyl Glycosides Related to the Pectic Rhamnogalacturonan I Polysaccharide

**DOI:** 10.3390/molecules23020327

**Published:** 2018-02-03

**Authors:** Alexandra N. Zakharova, Shahid I. Awan, Faranak Nami, Charlotte H. Gotfredsen, Robert Madsen, Mads H. Clausen

**Affiliations:** 1 Center for Nanomedicine and Theranostics, Technical University of Denmark, Kemitorvet 207, DK-2800 Kgs. Lyngby, Denmark; alex.zakharova@gmail.com (A.N.Z.); awanishahid@gmail.com (S.I.A.); fnam@kemi.dtu.dk (F.N.); 2 Department of Chemistry, Technical University of Denmark, Kemitorvet 207, DK-2800 Kgs. Lyngby, Denmark; chg@kemi.dtu.dk (C.H.G.); rm@kemi.dtu.dk (R.M.)

**Keywords:** pectin, rhamnogalacturonan I, branched RG-I, oligosaccharide synthesis

## Abstract

The synthesis of two protected tetrasaccharide pentenyl glycosides with diarabinan and digalactan branching related to the pectic polysaccharide rhamnogalacturonan I is reported. The strategy relies on the coupling of *N*-phenyl trifluoroacetimidate disaccharide donors to a common rhamnosyl acceptor. The resulting trisaccharide thioglycosides were finally coupled to an *n*-pentenyl galactoside acceptor to access the two protected branched tetrasaccharides.

## 1. Introduction

Pectins are the most structurally complex polysaccharides found in plant cell walls. These highly heterogeneous polysaccharides are fundamental components of the primary cell wall of plants where they modulate plant cell functions such as support, defense, signaling, and cell adhesion [1,2]. They also play a vital role in the food industry, serving as stabilizing and thickening agents in products such as jams, yogurt, and jellies [3]. Furthermore, it has been documented that they are useful in the production of biodegradable films, materials for biomedical implantation, and in drug delivery [4,5,6]. Rhamnogalacturonan I (RG-I) is one of the structural classes of pectic polysaccharides, along with homogalacturonan, rhamnogalacturonan II, xylogalacturonan, and apiogalacturonan [7]. The chemical structure of RG-I consists of a backbone with alternating units of α-linked l-rhamnose and d-galacturonic acid. The backbone has numerous branches at the C-4 position of the l-rhamnose residues including galactans, arabinans, or arabinogalactans. Galactans are mostly linear chains of β-(1→4)-linked d-galactose residues. Arabinans are chains of α-(1→5)-linked l-arabinofuranosides that are frequently branched at C-3 and sometimes at C-2. The most frequent form of arabinogalactan side chains is arabinogalactan I, which is a β-(1→4)-galactan with arabinan branches [8,9]. To the best of our knowledge, except for the synthesis of tri- and tetrasaccharide building blocks containing a single galactose unit as a side chain by Vogel and co-workers [10,11], branched RG-I fragments have not previously been addressed by chemical synthesis [12]. These structures are of great interest because they are useful for the study of pectin and the enzymes involved in both the biosynthesis and degradation of RG-I [1,2,7,13,14]. Herein, we report the synthesis of two protected tetrasaccharides with diarabinan and digalactan branching, designed for the assembly of larger RG-I oligosaccharides.

**Scheme 1:**
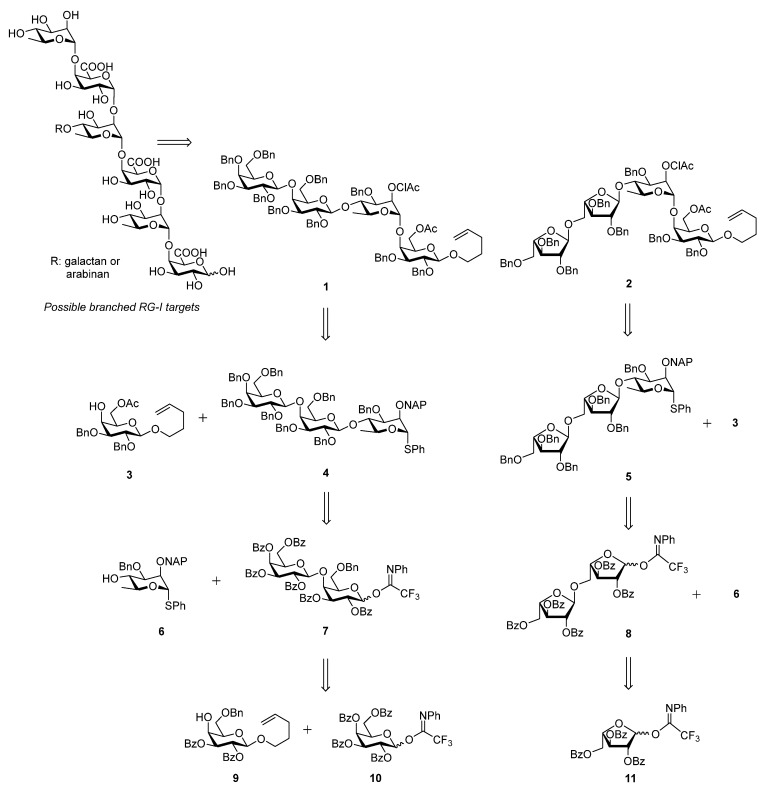
Retrosynthetic analysis of branched RG-I oligosaccharides **1** and **2**.

The retrosynthetic analysis of targets **1** and **2** is depicted in Scheme 1. We invisioned that both targets could be obtained in a linear synthetic strategy with the last glycosylation involving the same galactosyl acceptor **3**. We have previously succesfully used galactosides in the assembly of an unprotected hexasaccharide backbone fragment of RG-I using a post-glycosylation-oxidation strategy, which has also been successfully applied by others [15,16,17]. The two trisaccharides **4** and **5** could be assembled by employing the common acceptor **6** forming the disaccharidic backbone unit for both targets. The putative side chain consisting of digalactan **7** can be assembled by glycosylating galactose acceptor **9** with donor **10**. Likewise, diarabinan **8** can be derived from **11**.

## 2. Results and Discussion

**Scheme 2:**
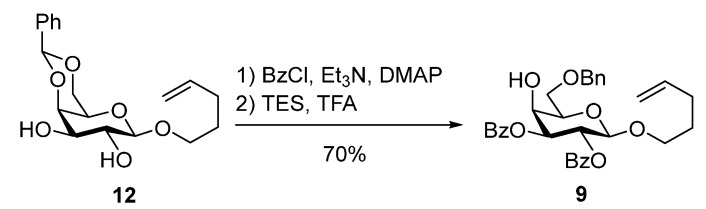
Synthesis of the *n*-pentenyl galactoside **9**.

Starting from β-d-galactose pentaacetate, galactosyl acceptor **3** was synthesized according to our previous work [18,19] and the diol **12** was accessed as reported earlier [20]. After masking the free hydroxyl groups of compound **12** as benzoyl esters a regioselective opening [21,22,23] of the benzylidene acetal resulted in acceptor **9** in 70% yield over two steps (Scheme 2).

**Scheme 3:**
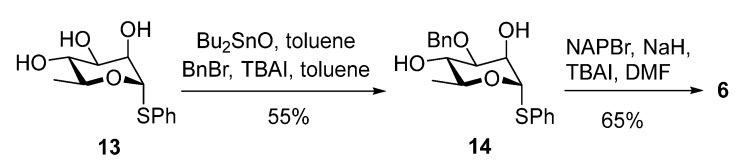
Synthesis of the rhamnosyl acceptor **6**.

The *N*-phenyl trifluoroacetimidate donor **10** was prepared from commercially available d-galactose in four steps [24]. Rhamnosyl acceptor **6** was obtained in two steps from l-rhamnose (Scheme 3). Triol **13 [25]** was transformed into the diol **14 [26]** by a tin-mediated regioselective benzylation of the C-3 hydroxyl group. The higher acidity of the C-2 alcohol was exploited to protect it as a NAP-ether. In the literature [27], there is an example of a selective benzylation of a C-2 hydroxyl group in a similar rhamnose derivative under phase-transfer conditions, however, we observed low yields using the same reaction conditions due to the formation of the regioisomer. The NAP-protected rhamnoside **6** was instead synthesized by reacting the diol **14** with NAPBr in the presence of sodium hydride and TBAI resulting in **6** in 65% yield (Scheme 3).

**Scheme 4:**
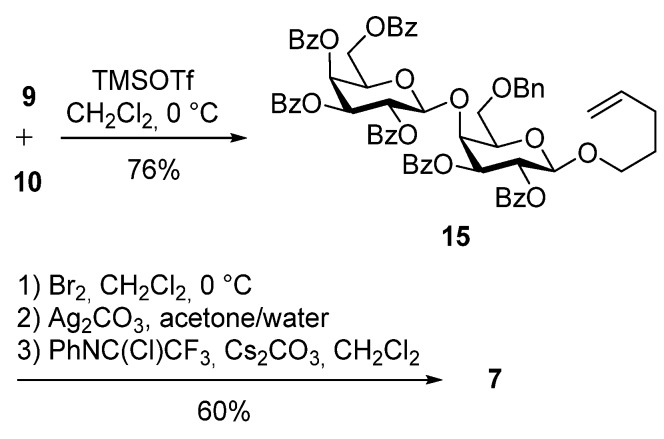
Synthesis of the digalactan *N*-phenyl trifluoroacetimidate donor **7**.

The synthesis of the digalactan *N*-phenyl trifluoroacetimidate donor **7** started by TMSOTf-promoted glycosylation of acceptor **9** with the perbenzoylated donor **10**. The reactants were mixed at −40 °C and then immediately warmed to 0 °C. The reaction mixture was left stirring at this temperature for 3 h resulting in disaccharide **15** in 76% yield. The hydrolysis of the *n*-pentenyl group was achieved in two steps: first **15** was titrated with bromine at 0 °C, and then the resulting bromide was treated directly with silver(I) carbonate in a mixture of acetone and water. This approach afforded the corresponding hemiacetal, which was further reacted with *N*-phenyl trifluoroacetimidoyl chloride in the presence of cesium carbonate to give the digalactan donor **7** in 60% yield over three steps (Scheme 4).

**Scheme 5:**
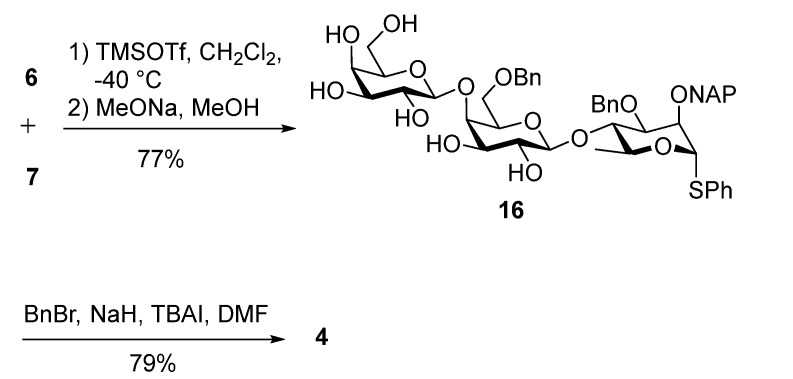
Synthesis of digalactan-containing trisaccharide donor **4**.

Rhamnose acceptor **6** was then glycosylated with donor **7** followed by a Zemplén deacylation yielding the trisaccharide **16** in 77% yield over two steps. Global benzylation gave trisaccharide donor **4** in 79% yield (Scheme 5).

Having prepared the trisaccharide thiophenyl donor **4**, we investigated the coupling with galactose acceptor **3**. We examined different promoters: NIS/TESOTf, NIS/Yb(OTf)_3_, MeOTf, Ph_2_SO/Tf_2_O, DMTST, Me_2_S_2_/Tf_2_O, but in all cases, the yield of the desired product was unsatisfactory. Furthermore, screening of these reaction conditions with monomeric building blocks mainly generated a *C*-glycoside through an intramolecular cyclization of the NAP moiety, as also observed by Crich et al. [28]. Clearly, the NAP-group had to be replaced to avoid the formation of the cyclized by-product, and the chloroacetyl (ClAc) ester was chosen as it can be removed selectively in the presence of the C-6 acetyl group.

**Scheme 6:**
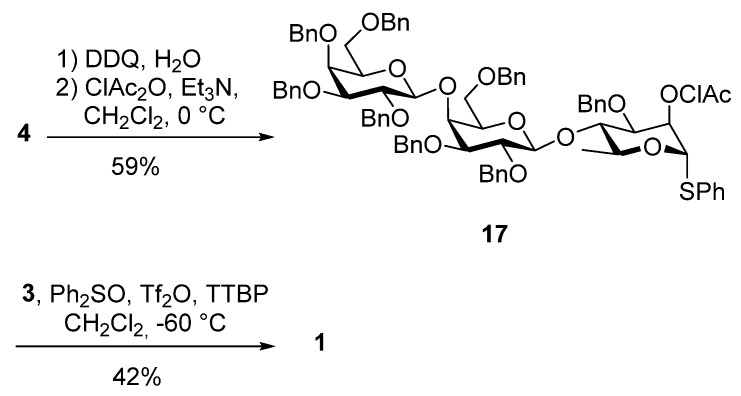
Synthesis of tetrasaccharide **1**.

Treatment of **4** with DDQ in the presence of water in a mixture of CH_2_Cl_2_ and methanol afforded the corresponding alcohol. The free hydroxyl group was then protected as a chloroacetyl ester by treating it with chloroacetic anhydride under basic conditions (59% over two steps). A mixture of donor **17** and galactose acceptor **3** in CH_2_Cl_2_ was treated with Ph_2_SO/Tf_2_O and giving the target tetrasaccharide **1** in 42% yield (Scheme 6).

**Scheme 7:**
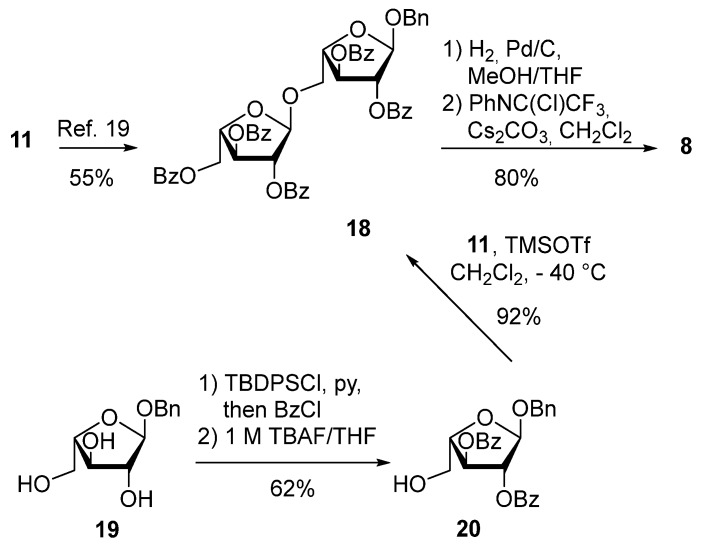
Synthesis of the diarabinan *N*-phenyl trifluoroacetimidate donor **8**.

Next, we turned our attention to the synthesis of tetrasaccharide **2**. Diarabinan *N*-phenyl trifluoroacetimidate donor **8** was prepared in six steps starting from the arabinose donor **11** [29] similar to previous work by Varrot and coworkers [30] (Scheme 7). 

**Scheme 8:**
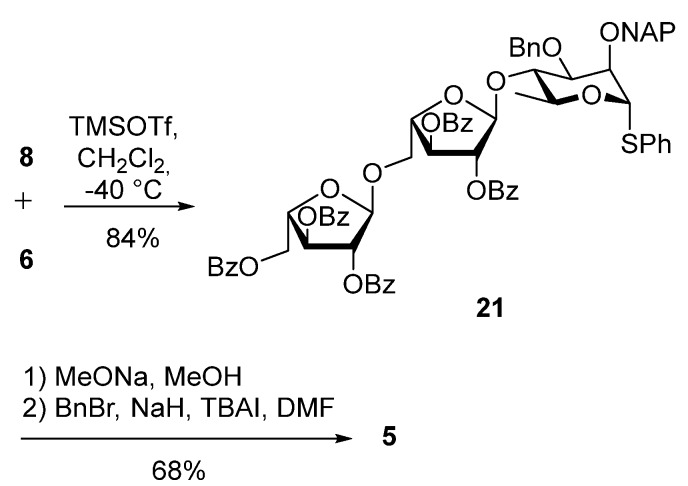
Synthesis of the diarabinan-containing trisaccharide donor **5**.

We also explored an alternative route to the disaccharide **18** (Scheme 7). Triol **19** [30] was transformed into the fully protected arabinose derivative through a two-step, one-pot protocol. Subsequently, the TBDPS-group was removed by treatment with TBAF (62% over two steps). The resulting alcohol **20** was coupled with donor **11** to give **18** in 92% yield. The benzyl group was used as temporary protection of the anomeric position in **18**. Catalytic hydrogenolysis provided the corresponding hemiacetal, which was transformed into the target disaccharide donor **8** in 80% over two steps. The synthesis of the diarabinan-containing trisaccharide **5** is shown in Scheme 8. TMSOTf-mediated coupling of **8** with rhamnose acceptor **6** afforded trisaccharide **21** in 84% yield. The presence of the participating benzoyl group at the C-2 position of donor **8** ensured the formation of the α-glycosidic linkage. The benzoyl esters of **21** were exchanged with benzyl protective groups in two steps (68% yield) giving trisaccharide donor **5**.

**Scheme 9:**
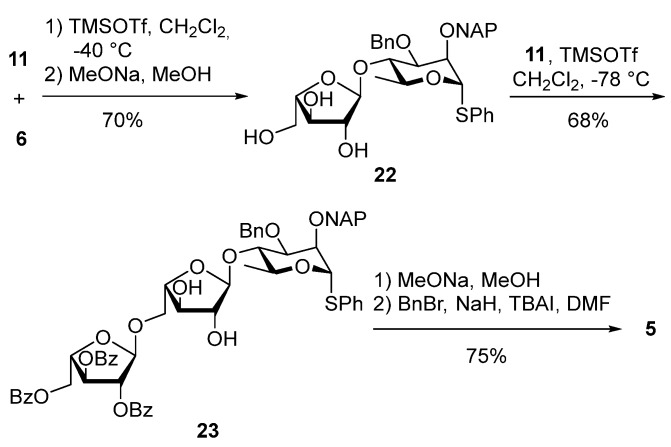
Synthesis of trisaccharide **5** by an alternative approach.

For the synthesis of the diarabinan-containing trisaccharide donor **5**, an alternative approach to the one described above also gave excellent results, as shown in Scheme 9. The rhamnose acceptor **6** was glycosylated with arabinose donor **11** followed by deacylation under Zemplén conditions providing triol **22** (70% yield over two steps). Selective glycosylation of the primary alcohol using donor **11** afforded the partially protected trisaccharide **23** in 68% yield. Protecting group manipulations gave **5** in 75% yield over two steps. This approach allowed us to reach trisaccharide **5** in five steps from **6** and **11**.

**Scheme 10:**
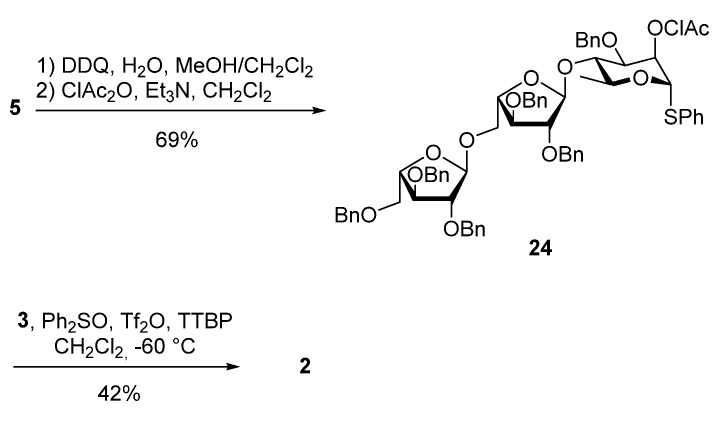
Synthesis of the tetrasaccharide **2**.

As discussed previously, the NAP-group was exchanged with a chloroacetyl ester to give **24** in 69% yield over two steps. Final glycosylation of galactosyl acceptor **3** with trisaccharide donor **24** was performed, using Ph_2_SO/Tf_2_O as a promoter at −60 °C, resulting in the desired tetrasaccharide **2** in 42% yield (Scheme 10).

## 3. Materials and Methods

### 3.1. General Information

Starting materials, reagents, and solvents were purchased from commercial suppliers and have been used without further purification. All solvents were HPLC-grade. Anhydrous solvents were obtained from Innovative Technology PS-MD-7 Pure-solv solvent purification system (Innovative Technology, Pewaukee, WI, USA). The reactions were carried out in flame-dried glassware under an inert atmosphere unless stated otherwise. Thin-layer chromatography (TLC) was performed on Merck (Merck, Darmstadt, Germany) Aluminium Sheets pre-coated with silica, C-60 F254 plates. Compounds were visualized by charring after dipping in CAM stain (Ce(SO_4_)_2_ (1.6 g) and (NH_4_)_6_Mo_7_O_24_ (4 g) in 10% sulphuric acid (200 mL). Eluent systems are specified for each R*_f_* value, and ratios are given as volume ratios. Evaporation of solvents was performed with a VWR International Laborota 400 under reduced pressure (in vacuo) at temperatures ranging between 35–55 °C. Trace solvent was removed under reduced pressure using a vacuum pump (Edwards, Burgess Hill, UK). Flash chromatography was performed using Matrex 60 Å silica gel (35–70 μm, Merck, Darmstadt, Germany) as the stationary phase by the general procedure developed by Still et al. [31]*.* The eluent system is specified by the protocol for each synthesis. Eluent ratios are given as volume ratios. NMR-spectra were recorded on a Varian Mercury 300 B (Varian, Palo Alto, CA, USA), Bruker Ascend 400, Bruker Avance 800 MHz, Bruker DQX 400 or Bruker AC 500 spectrometer (Bruker, Billerica, MA, USA). Chemical shifts (δ) are reported in ppm and coupling constants in Hz. Solvents used for NMR experiments were CDCl_3_, CD_3_OD, D_2_O or *d*_6_-DMSO and their resonances were used as internal standards. Most of the compounds have been characterized by NMR. 

### 3.2. General Procedures

**General Procedure I for Glycosylation with *N*-Phenyl Trifluoroacetimidate Glycosyl Donors**

A mixture of the donor (1.2 equiv.) and the acceptor (1.0 equiv.) was co-evaporated with toluene and subjected to high vacuum for 2 h. The mixture was dissolved in anhydrous CH_2_Cl_2_ (*c*. 0.1–0.01 M) and cooled to −40 °C. TMSOTf (0.1 equiv.) was added, and the reaction mixture was stirred at −40 °C until TLC showed completion of the reaction (10–30 min). The reaction mixture was quenched by addition of Et_3_N, evaporated and purified by flash column chromatography.

**General Procedure II for Preparation of the *N*-Phenyl Trifluoroacetimidate Glycosyl Donors**

Hemiacetal (1.0 equiv.) was dissolved in CH_2_Cl_2_ (*c*. 0.1–0.01 M). PhNC(Cl)CF_3_ (2.0 equiv.) and Cs_2_CO_3_ (2.0 equiv.) were added and the reaction mixture was stirred at 20 °C until TLC showed completion of the reaction (2–5 h). The reaction mixture was filtered through a plug of Celite, concentrated, and the residue was purified by flash column chromatography.

**General Procedure III for Removal of Acyl Protective Groups (Zemplén Conditions)**

The starting material was dissolved in MeOH (*c*. 0.1–0.01 M) or, in a 1:1 mixture of MeOH and THF. A freshly prepared 1 M NaOMe solution (0.1 equiv.) in MeOH was added. The reaction mixture was stirred at 20 °C until TLC showed the full conversion (1–24 h). The reaction mixture was then quenched by addition of Amberlite IR-120 (H^+^). The resin was filtered off, and the filtrate was concentrated, and purified by flash column chromatography.

**General Procedure IV for Benzylation of Hydroxyl Groups**

To a solution of the starting material (1.0 equiv.) in DMF, BnBr (1.2 equiv. for 1-OH) and TBAI (0.01 equiv.) were added, and the mixture was cooled in an ice bath. NaH (1.2 equiv. for 1-OH) was added and the mixture was stirred at 20 °C for 15 h and then quenched by addition of MeOH. The reaction mixture was partially concentrated, diluted with EtOAc and washed with water and brine. The organic phase was dried over Na_2_SO_4_, concentrated and purified by flash column chromatography. 

**General Procedure V for Removal of the 2-Naphthylmethyl (NAP) Group**

The protected saccharide (1.0 equiv.) was dissolved in a 4:1 mixture of CH_2_Cl_2_ and MeOH. Water (2%) was added followed by addition of DDQ (1.4 equiv.). The reaction mixture was stirred at 20 °C until TLC showed completion of the reaction (2–5 h). The reaction mixture was diluted with CH_2_Cl_2_ and washed with sat. NaHCO_3_. The combined aqueous phases were extracted with CH_2_Cl_2_. The combined organic phases were dried over Na_2_SO_4_, filtered, concentrated and purified by flash chromatography.

**General Procedure VI for Introducing a Chloroacetyl (ClAc) Group**

Alcohol (1.0 equiv.) was dissolved in anhydrous CH_2_Cl_2_ and cooled in an ice bath. Et_3_N (2.0 equiv.) was added followed by addition of ClAc_2_O (1.1 equiv.). The reaction mixture was stirred at 0 °C for 2 h, then warmed up to 20 °C, diluted with CH_2_Cl_2_ and washed with 0.1 M HCl. The organic phase was dried over Na_2_SO_4_, concentrated and purified by flash column chromatography.

**General Procedure VII the Ph_2_SO/Tf_2_O-Promoted Glycosylation**

A mixture of the donor (1.2 equiv.), Ph_2_SO (1.2 equiv.) and TTBP (1.2 equiv.) was co-evaporated with toluene and subjected to high vacuum for 2 h. The mixture was dissolved in anhydrous CH_2_Cl_2_ and cooled to −60 °C. Tf_2_O (1.3 equiv.) was added, and the reaction mixture was stirred at −60 °C for 5 min. Afterwards, a solution of the acceptor (1.0 equiv.) in anhydrous CH_2_Cl_2_ was added. The mixture was warmed to −40 °C over 2 h and Et_3_N was added. The mixture was diluted with CH_2_Cl_2_ and washed with brine, dried over Na_2_SO_4_, concentrated and purified by flash chromatography.

**Pent-4-enyl 2,3-di-*O*-benzoyl-6-*O*-benzyl-β-d-galactopyranoside 9**

Compound **12** (4 g, 11.90 mmol) was dissolved in CH_2_Cl_2_ (100 mL) followed by the addition of Et_3_N (5.0 mL, 35.70 mmol), DMAP (9 mg, 0.39 mmol) and benzoyl chloride (3.5 mL, 29.75 mmol). The reaction mixture was stirred until TLC revealed full conversion (3 h). The reaction was quenched with MeOH (10 mL), washed with water (2 × 100 mL), dried over MgSO_4_ and concentrated. The product was purified by flash chromatography (toluene/EtOAc 19:1) to afford the corresponding product as a white powder. Yield 6.0 g (94%). R*_f_* 0.44 (9:1 toluene/EtOAc). ^1^H-NMR (400 MHz, CDCl_3_) δ 7.91 (m, 4H), 7.54–7.36 (m, 4H), 7.35–7.24 (m, 7H), 5.79 (dd, *J* = 10.4, 8.0 Hz, 1H), 5.58 (ddt, *J* = 16.2, 10.9, 6.7 Hz, 1H), 5.47 (s, 1H), 5.29 (dd, *J* = 10.4, 3.6 Hz, 1H), 4.76–4.69 (m, 2H), 4.66 (d, *J* = 8.0 Hz, 1H), 4.51 (dd, *J* = 3.6, 1.0 Hz, 1H), 4.32 (dd, *J* = 12.4, 1.6 Hz, 1H), 4.06 (dd, *J* = 12.4, 1.8 Hz, 1H), 3.89 (dt, *J* = 9.6, 6.1 Hz, 1H), 3.59 (m, 1H), 3.46 (ddd, *J* = 9.6, 7.3, 6.2 Hz, 1H), 1.99–1.79 (m, 2H), 1.68–1.42 (m, 2H). ^13^C-NMR (101 MHz, CDCl_3_) δ 166.3, 165.3, 138.0, 137.6, 133.4, 133.1, 130.0, 129.8, 129.8, 129.2, 129.0, 128.5, 128.4, 128.2, 126.4, 114.8, 101.3, 101.0, 73.7, 72.9, 69.2, 69.1, 68.9, 66.6, 29.9, 28.6.

Trifluoroacetic acid (7.0 mL, 91.88 mmol) was added dropwise to a solution of the above mentioned compound (10.0 g, 18.37 mmol) and triethylsilane (14.7 mL, 91.88 mmol) in CH_2_Cl_2_ (75 mL) at 0 °C. When the addition was complete (10 min), the reaction was heated to 22 °C and stirred for 5 h. The mixture was diluted with EtOAc and washed with sat. aq. NaHCO_3_ and brine, dried over MgSO_4_, filtered and concentrated. The product was purified by flash chromatography (19:1 toluene/EtOAc) to afford **9** as a white amorphous solid. Yield: 7.1 (72%). R*_f_* 0.38 (9:1 toluene/EtOAc). ^1^H-NMR (400 MHz, CDCl_3_) δ 7.95–7.85 (m, 4H), 7.47–7.38 (m, 2H), 7.33–7.19 (m, 9H), 5.75–5.65 (dd, *J* = 9.5 Hz, 1H), 5.57 (ddt, *J* = 15.7, 11.2, 6.6 Hz, 1H), 5.21 (dt, *J* = 9.5, 1.6 Hz, 1H), 4.76–4.73 (m, 2H), 4.60 (d, *J* = 9.5 Hz, 1H), 4.52 (m, 2H), 4.31–4.28 (m, 1H), 3.85 (dt, *J* = 9.7, 6.2 Hz, 1H), 3.80–3.69 (m, 3H), 3.51–3.39 (m, 1H), 1.98–1.81 (m, 2H), 1.62–1.45 (m, 2H).^13^C-NMR (101 MHz, CDCl_3_) δ 165.9, 165.3, 137.9, 137.6, 137.6, 133.3, 133.0, 129.8, 129.6, 129.1, 128.5, 128.4, 128.3, 127.8, 127.8, 114.7, 101.5, 74.4, 74.4, 73.8, 73.3, 69.7, 69.2, 69.1, 68.1, 29.8, 28.6.

**Phenyl 3-*O*-benzyl-2-*O*-(2-naphthylmethyl)-1-thio-α-l-rhamnopyranoside 6**

To a solution of diol **14** (700 mg, 2.0 mmol) in DMF (6 mL) NAPBr (490 mg, 2.2 mmol) and TBAI (75 mg, 0.2 mmol) were added and the mixture was cooled in an ice bath. NaH (90 mg, 2.2 mmol, 60% in oil) was added and the mixture was stirred at 20 °C for 12 h and then quenched by addition of MeOH. The reaction mixture was partially concentrated, diluted with EtOAc and washed with water and brine. The combined water phase was extracted with EtOAc. The combined organic phase was dried over Na_2_SO_4_, concentrated and purified by flash chromatography (19:1 toluene/EtOAc) to afford **6** as a colorless oil. Yield 640 mg (65%). R*_f_* 0.22 (toluene/EtOAc 20:1). ^1^H-NMR (300 MHz, CDCl_3_) δ 7.75–7.66 (m, 4H), 7.41–7.38 (m, 3H), 7.60–7.16 (m, 10H), 5.49 (d, *J* = 1.2 Hz, 1H), 4.80 (d, *J* = 12.4 Hz, 1H), 4.64 (d, *J* = 12.4 Hz, 1H), 4.49 (d, *J* = 11.7 Hz, 1H), 4.37 (d, *J* = 11.7 Hz, 1H), 4.06–4.01 (m, 1H), 4.00–3.98 (m, 1H), 3.76 (t, *J* = 9.4 Hz, 1H), 3.58 (dd, *J* = 9.5, 3.0 Hz, 1H), 2.26 (s, 1H), 1.30 (d, *J* = 6.1 Hz, 3H); ^13^C-NMR (75 MHz, CDCl_3_) δ 137.5, 135.0, 134.4, 133.0, 132.9, 131.1, 128.9, 128.4, 128.1, 127.8, 127.8, 127.5, 127.2, 126.7, 126.0, 125.8, 85.7, 79.5, 75.4, 71.9, 71.7, 71.4, 69.6, 17.6.

**Pent-4-enyl 2,3,4,6-tetra-*O*-benzoyl-β-d-galactopyranosyl-(1→4)-2,3-di*-O-*benzoyl-6*-O-*benzyl-β-d-galactopyranoside 15**

Prepared from **9** and **10** according to the General Procedure I. White amorphous solid, 76%. R*_f_* 0.35 (toluene/EtOAc 10:1). ^1^H-NMR (300 MHz, CDCl_3_) δ 8.00–7.93 (m, 4H), 7.91–7.69 (m, 8H), 7.49–7.12 (m, 23H), 5.80–5.76 (m, 2H), 5.58–5.45 (m, 2H), 5.43–5.28 (m, 2H), 4.98 (d, *J* = 7.9 Hz, 1H), 4.74 (d, *J* = 1.1 Hz, 1H), 4.69 (m, 2H), 4.58 (d, *J* = 7.5 Hz, 1H), 4.54 (d, *J* = 5.6 Hz, 1H), 4.47 (d, *J* = 2.8 Hz, 1H), 4.32 (dd, *J* = 11.2, 6.5 Hz, 1H), 4.22 (dd, *J* = 11.3, 6.6 Hz, 1H), 3.94 (t, *J* = 6.5 Hz, 1H), 3.88–3.74 (m, 4H), 3.47–3.34 (m, 1H), 1.89–1.78 (m, 2H), 1.56–1.40 (m, 2H); ^13^C-NMR (75 MHz, CDCl_3_) δ 166.7, 166.6, 166.4, 166.2, 165.3, 138.8, 138.8, 134.4, 134.1, 133.6, 130.8, 130.7, 130.6, 130.5, 130.5, 130.5, 130.4, 130.3, 130.0, 129.8, 129.5, 129.4, 129.2, 129.1, 129.0, 129.0, 128.5, 128.5, 115.4, 102.0, 101.7, 74.9, 74.6, 74.5, 74.0, 72.6, 72.0, 70.9, 70.4, 70.3, 69.3, 68.9, 62.5, 30.6, 29.3.

**2,3,4,6-Tetra-*O*-benzoyl-β-d-galactopyranosyl-(1→4)-2,3-di*-O-*benzoyl-6*-O-*benzyl-d-galactopyranosyl *N*-phenyl trifluoroacetimidate 7**

The hemiacetal was prepared from pentenyl glycoside **15** as described for a similar compound in the literature [32] and it was converted into **7** according to the General Procedure II. White foam, 85%. The product was verified by NMR spectroscopy as an α/β mixture and was used in the subsequent glycosylation without further characterization.

**Phenyl β-d-galactopyranosyl-(1→4)-6-*O*-benzyl-β-d-galactopyranosyl-(1→4)-3-*O*-benzyl-2-*O*-(2-naphthylmethyl)-1-thio-α-l-rhamnopyranoside 16**

Compound **16** was prepared from **6** and **7** according to the General Procedure I and subjected to conditions from General Procedure III without further purification. White amorphous solid, 77%. R*_f_* 0.25 (CH_2_Cl_2_/MeOH 10:1). ^1^H-NMR (300 MHz, CDCl_3_) δ 7.73–7.52 (m, 5H), 7.42–7.01 (m, 17H), 5.34 (s, 1H), 4.66–4.24 (m, 8H), 4.05–3.25 (m, 16H), 1.24 (d, *J* = 5.0 Hz, 3H); ^13^C-NMR (75 MHz, CDCl_3_) δ 138.0, 137.6, 135.0, 134.3, 133.0, 132.9, 131.1, 128.9, 128.5, 128.4, 128.3, 128.2, 127.8, 127.6, 127.4, 127.2, 126.8, 126.0, 125.9, 125.8, 105.7, 104.1, 85.5, 79.5, 79.2, 75.9, 74.5, 73.9, 73.6, 73.0, 72.6, 72.4, 72.2, 72.0, 68.9, 68.6, 68.3, 61.0, 17.8.

**Phenyl 2,3,4,6-tetra-*O*-benzyl-β-d-galactopyranosyl-(1→4)-2,3,6-tri-*O-*benzyl-β-d-galactopyranosyl-(1→4)-3-*O*-benzyl-2-*O*-(2-naphthylmethyl)-1-thio-α-l-rhamnopyranoside 4**

Prepared from **16** according to the General Procedure IV. White amorphous solid, 78%. R*_f_* 0.27 (toluene/EtOAc 20:1). ^1^H-NMR (400 MHz, CDCl_3_) δ 7.73–7.55 (m, 4H), 7.42–7.31 (m, 6H), 7.29–7.05 (m, 45H), 5.37 (d, *J* = 1.7 Hz, 1H), 5.02 (d, *J* = 11.0 Hz, 1H), 4.96 (d, *J* = 7.6 Hz, 1H), 4.89 (d, *J* = 11.6 Hz, 1H), 4.82 (d, *J* = 7.7 Hz, 1H), 4.76–4.59 (m, 6H), 4.54 (d, *J* = 11.5 Hz, 1H), 4.49 (d, *J* = 11.6 Hz, 1H), 4.46 (s, 1H), 4.34–4.25 (m, 4H), 4.16 (d, *J* = 11.2 Hz, 1H), 4.10–3.99 (m, 1H), 3.93 (t, *J* = 9.1 Hz, 1H), 3.83–3.76 (m, 2H), 3.74–3.63 (m, 4H), 3.57–3.50 (m, 2H), 3.50–3.44 (m, 2H), 3.43–3.32 (m, 5H), 1.27 (d, *J* = 6.1 Hz, 1H); ^13^C-NMR (101 MHz, CDCl_3_) δ 139.2, 139.0, 138.9, 138.8, 138.5, 138.5, 138.2, 137.9, 135.2, 134.5, 133.1, 132.9, 131.4, 128.9, 128.3, 128.3, 128.2, 128.2, 128.2, 128.1, 128.1, 128.0, 128.0, 127.9, 127.7, 127.7, 127.7, 127.6, 127.5, 127.5, 127.4, 127.3, 127.3, 127.2, 127.2, 127.1, 126.8, 126.0, 126.0, 125.8, 102.8, 102.2, 85.8, 82.4, 81.8, 80.4, 80.2, 79.7, 76.5, 76.1, 75.3, 74.6, 74.6, 74.3, 73.4, 73.4, 73.3, 73.1, 73.1, 72.3, 72.3, 72.0, 69.3, 69.1, 69.0, 68.6, 17.9.

**Phenyl 2,3,4,6-tetra-*O*-benzyl-β-d-galactopyranosyl-(1→4)-2,3,6-tri*-O-*benzyl-β-d-galactopyranosyl-(1→4)-3-*O*-benzyl-1-thio-α-l-rhamnopyranoside S1**

Prepared from **4** according to the General Procedure V. White amorphous solid, 73%. R*_f_* 0.23 (toluene/EtOAc 10:1). ^1^H-NMR (400 MHz, CDCl_3_) δ 7.39–7.31 (m, 4H), 7.27–7.07 (m, 39H), 7.04 (d, *J* = 7.8 Hz, 2H), 5.39 (d, *J* = 1.3 Hz, 1H), 4.99 (d, *J* = 11.0 Hz, 1H), 4.92 (d, *J* = 7.6 Hz, 1H), 4.87 (d, *J* = 11.6 Hz, 1H), 4.74–4.66 (m, 3H), 4.65–4.52 (m, 5H), 4.46 (d, *J* = 11.6 Hz, 1H), 4.41 (s, 2H), 4.31 (d, *J* = 10.8 Hz, 1H), 4.28–4.18 (m, 4H), 4.12–4.02 (m, 1H), 4.01–3.98 (m, 1H), 3.77–3.71 (m, 2H), 3.71–3.61 (m, 4H), 3.53–3.41 (m, 2H), 3.40–3.29 (m, 4H), 2.24 (s, 1H), 1.20 (d, *J* = 6.2 Hz, 3H); ^13^C-NMR (101 MHz, CDCl_3_) δ 139.0, 138.9, 138.7, 138.5, 138.4, 137.8, 137.6, 131.3, 128.9, 128.3, 128.3, 128.2, 128.2, 128.1, 128.1, 128.1, 128.1, 128.0, 127.8, 127.8, 127.7, 127.7, 127.7, 127.4, 127.3, 127.3, 127.3, 127.2, 125.2, 102.9, 102.2, 86.8, 82.3, 81.8, 80.5, 80.3, 79.7, 76.1, 75.4, 74.6, 74.6, 74.2, 73.4, 73.4, 73.3, 73.1, 72.3, 72.1, 69.7, 69.4, 69.1, 68.7, 68.3, 17.7. Copies of NMR spectra in Supplementary Materials.

**Phenyl 2,3,4,6-tetra-*O*-benzyl-β-d-galactopyranosyl-(1→4)-2,3,6-tri*-O-*benzyl-β-d-galactopyranosyl-(1→4)-3-*O*-benzyl-2-*O*-chloroacetyl-1-thio-α-l-rhamnopyranoside 17**

Prepared from **S1** according to the General Procedure VI. Colorless oil, 80%, R*_f_* 0.49 (toluene/EtOAc 10:1). ^1^H-NMR (400 MHz, CDCl_3_) δ 7.42–7.31 (m, 4H), 7.27–7.04 (m, 39H), 7.02 (d, *J* = 7.4 Hz, 2H), 5.48–5.43 (m, 1H), 5.29 (d, *J* = 1.4 Hz, 1H), 5.01 (d, *J* = 11.0 Hz, 1H), 4.92 (d, *J* = 7.6 Hz, 1H), 4.87 (d, *J* = 11.6 Hz, 1H), 4.73 (d, *J* = 13.7 Hz, 1H), 4.72–4.68 (m, 2H), 4.66–4.55 (m, 5H), 4.47 (d, *J* = 11.6 Hz, 1H), 4.42 (d, *J* = 2.4 Hz, 2H), 4.29–4.20 (m, 4H), 4.16–4.07 (m, 2H), 3.85 (d, *J* = 2.0 Hz, 2H), 3.75 (d, *J* = 2.9 Hz, 1H), 3.72–3.64 (m, 5H), 3.54 (dd, *J* = 10.1, 5.9 Hz, 1H), 3.49–3.41 (m, 1H), 3.42–3.30 (m, 5H), 1.25 (d, *J* = 6.2 Hz, 3H); ^13^C-NMR (101 MHz, CDCl_3_) δ 166.6, 139.0, 138.9, 138.8, 138.6, 138.4, 138.3, 137.8, 137.6, 137.3, 133.3, 131.8, 129.0, 128.9, 128.6, 128.3, 128.2, 128.1, 128.1, 128.0, 128.0, 128.0, 128.0, 127.8, 127.8, 127.7, 127.6, 127.3, 127.2, 125.1, 102.6, 102.2, 85.6, 82.1, 81.7, 80.3, 79.6, 78.4, 75.9, 75.5, 74.5, 74.2, 73.4, 73.3, 73.3, 73.1, 73.0, 72.2, 72.1, 72.0, 69.7, 69.4, 68.6, 68.6, 40.7, 17.7.

**Pent-4-enyl 2,3,4,6-tetra-*O*-benzyl-β-d-galactopyranosyl-(1→4)-2,3,6-tri-*O*-benzyl-β-d-galactopyranosyl-(1→4)-3-*O*-benzyl-2-*O*-chloroacetyl-α-l-rhamnopyranosyl-(1→4)-6-*O*-acetyl-2,3-di-*O*-benzyl-β-d-galactopyranoside 1**

Prepared from **17** and **3** by following general procedure VII. Colorless oil, 42%. R*_f_* 0.14 (toluene/EtOAc 10:1). ^1^H-NMR (400 MHz, CDCl_3_) δ 7.37 (d, *J* = 6.6 Hz, 2H), 7.32–7.08 (m, 48H), 5.80–5.67 (m, 1H), 5.42 (dd, *J* = 3.0, 2.1 Hz, 1H), 5.11 (d, *J* = 1.6 Hz, 1H), 5.02 (d, *J* = 11.0 Hz, 1H), 4.94 (dd, *J* = 17.2, 1.7 Hz, 1H), 4.94–4.84 (m, 4H), 4.76–4.67 (m, 10H), 4.67–4.45 (m, 4H), 4.43 (d, *J* = 2.9 Hz, 2H), 4.35–4.24 (m, 5H), 4.19 (d, *J* = 2.2 Hz, 1H), 4.12 (dd, *J* = 11.1, 6.5 Hz, 1H), 4.04 (d, *J* = 9.6 Hz, 1H), 3.95 (d, *J* = 2.2 Hz, 1H), 3.93–3.88 (m, 1H), 3.85–3.58 (m, 8H), 3.58–3.31 (m, 10H), 2.16–2.05 (m, 2H), 1.92 (s, 3H), 1.77–1.68 (m, 2H), 1.21 (d, *J* = 6.1 Hz, 3H); ^13^C-NMR (101 MHz, CDCl_3_) δ 170.4, 166.1, 139.1, 139.0, 138.9, 138.7, 138.6, 138.5, 138.2, 137.9, 137.8, 137.6, 128.9, 128.4, 128.3, 128.3, 128.2, 128.2, 128.1, 128.1, 128.1, 128.0, 128.0, 128.0, 127.9, 127.8, 127.7, 127.6, 127.5, 127.4, 127.4, 127.3, 127.3, 127.2, 114.9, 104.0, 102.7, 102.4, 99.0, 82.1, 81.8, 81.3, 80.3, 79.6, 78.6, 77.8, 75.6, 75.5, 75.0, 74.6, 74.3, 73.4, 73.4, 73.4, 73.1, 73.1, 72.8, 72.4, 72.0, 71.4, 70.4, 69.8, 69.7, 68.7, 68.1, 62.6, 40.8, 30.1, 28.8, 20.7, 17.9.

**2,3,5-Tri-*O*-benzoyl-α-l-arabinofuranosyl-(1→5)-2,3-di-*O*-benzoyl-l-arabinofuranosyl *N*-phenyl trifluoroacetimidate 8**

Prepared from the hemiacetal [19] according to the General Procedure II. White amorphous solid, 87%. The product was verified by NMR spectroscopy as an α/β mixture and was used in the subsequent glycosylation without further characterization. 

**Phenyl 2,3,5-tri-*O*-benzoyl-α-l-arabinofuranosyl-(1→5)-2,3-di-*O*-benzoyl-α-l-arabinofuranosyl-(1→4)-3-*O*-benzyl-2-*O*-(2-naphthylmethyl)-1-thio-α-l-rhamnopyranoside 21**

Prepared from **8** and **6** according to the General Procedure I. White amorphous solid, 84%. R*_f_* 0.40 (toluene/EtOAc 20:1). ^1^H-NMR (300 MHz, CDCl_3_) δ 7.95–7.81 (m, 10H), 7.66–7.61 (m, 1H), 7.57 (m, 3H), 7.46–7.06 (m, 24H), 7.02 (d, *J* = 7.2 Hz, 1H), 6.98–6.90 (m, 3H), 5.82 (s, 1H), 5.55 (bs, 1H), 5.50 (s, 1H), 5.47 (d, *J* = 4.7 Hz, 1H), 5.40 (bs, 1H), 5.35 (s, 1H), 4.75–4.57 (m, 5H), 4.56–4.36 (m, 4H), 4.15–3.97 (m, 3H), 3.92–3.76 (m, 3H), 1.29 (d, *J* = 5.8 Hz, 3H); ^13^C-NMR (75 MHz, CDCl_3_) δ 166.0, 165.6, 165.4, 165.0, 164.9, 137.5, 135.1, 134.5, 133.3, 133.2, 133.1, 132.9, 132.8, 131.0, 129.7, 129.6, 129.6, 129.5, 129.1, 128.9, 128.8, 128.7, 128.3, 128.2, 128.1, 127.7, 127.7, 127.5, 127.4, 127.1, 126.6, 125.9, 125.8, 125.8, 106.6, 105.9, 85.9, 82.5, 81.8, 81.5, 81.1, 80.2, 77.6, 77.6, 75.9, 75.5, 72.2, 71.8, 68.7, 66.4, 63.5, 18.0.

**Phenyl α-l-arabinofuranosyl-(1→5)-** **α-l-arabinofuranosyl-(1→4)-3-*O*-benzyl-2-*O*-(2-naphthylmethyl)-1-thio-α-l-rhamnopyranoside S2**

Prepared from **21** according to the General Procedure III. White amorphous solid, 87%. R*_f_* 0.37 (CH_2_Cl_2_/MeOH 10:1). ^1^H-NMR (300 MHz, CDCl_3_) δ 7.72–7.60 (m, 3H), 7.54 (bs, 1H), 7.39–7.30 (m, 4H), 7.26–7.13 (m, 7H), 7.12–7.07 (m, 2H), 5.38 (s, 1H), 5.32 (s, 1H), 4.93 (s, 1H), 4.66 (d, *J* = 12.4 Hz, 1H), 4.58 (d, *J* = 12.5 Hz, 1H), 4.36 (bs, 1H), 4.10 – 3.74 (m, 11H), 3.72–3.50 (m, 4H), 1.23 (d, *J* = 5.9 Hz, 3H); ^13^C-NM**R** (101 MHz, CDCl_3_) δ 137.5, 134.8, 134.1, 133.0, 132.9, 131.4, 128.9, 128.5, 128.3, 128.1, 127.9, 127.8, 127.6, 127.3, 127.0, 126.1, 126.0, 125.9, 109.0, 107.8, 85.6, 85.3, 83.5, 81.6, 80.6, 79.3, 77.9, 76.8, 76.0, 75.8, 72.0, 71.9, 68.8, 66.3, 61.7, 17.9. Copies of NMR spectra in Supplementary Materials.

**Phenyl 2,3,5-tri-*O*-benzyl-α-l-arabinofuranosyl-(1→5)-2,3-di-*O*-benzyl-α-l-arabinofuranosyl-(1→4)-3-*O*-benzyl-2-*O*-(2-naphthylmethyl)-1-thio-α-l-rhamnopyranoside 5**

Prepared from **S2** according to the General Procedure IV. White amorphous solid, 78%. R*_f_* 0.32 (toluene/EtOAc 20:1). ^1^H-NMR (300 MHz, CDCl_3_) δ 7.72–7.64 (m, 1H), 7.63–7.56 (m, 3H), 7.39–7.34 (m, 3H), 7.31–7.25 (m, 4H), 7.24–7.11 (m, 29H), 7.03–6.95 (m, 2H), 5.55 (s, 1H), 5.45 (bs, 1H), 5.08 (s, 1H), 4.74 (d, *J* = 12.5 Hz, 1H), 4.63 (d, *J* = 12.5 Hz, 1H), 4.60 (s, 1H), 4.49–4.40 (m, 8H), 4.37–4.20 (m, 3H), 4.19–4.00 (m, 3H), 3.97 (dd, *J* = 5.0, 2.1 Hz, 5H), 3.87–3.73 (m, 3H), 3.64–3.45 (m, 3H), 1.30 (d, *J* = 5.8 Hz, 3H); ^13^C-NMR (75 MHz, CDCl_3_) δ 138.0, 137.9, 137.8, 137.5, 137.5, 135.2, 134.5, 133.0, 132.9, 131.2, 128.9, 128.5, 128.3, 128.3, 128.2, 128.2, 128.1, 127.9, 127.8, 127.7, 127.7, 127.6, 127.6, 127.5, 127.5, 127.2, 127.1, 126.9, 126.6, 126.0, 125.9, 125.8, 107.0, 106.3, 88.4, 88.0, 85.8, 83.4, 80.8, 80.6, 80.2, 76.1, 75.5, 73.3, 72.1, 72.0, 71.8, 71.5, 71.1, 69.5, 69.0, 66.0, 65.2, 17.9.

**Phenyl α-l-arabinofuranosyl-(1→4)-3-*O*-benzyl-2-*O*-(2-naphthylmethyl)-1-thio-α-l-rhamnopyranoside 22**

Acceptor **6** was glycosylated with donor **11** according to the General Procedure I. The product was taken directly to the Zemplén deacylation according to General Procedure III. White amorphous solid, 70% over 2 steps. R*_f_* 0.28 (CH_2_Cl_2_/MeOH 10:1). ^1^H-NMR (400 MHz, CDCl_3_) δ 7.74–7.59 (m, 4H), 7.41–7.34 (m, 3H), 7.28–7.18 (m, 7H), 7.16–7.09 (m, 3H), 5.41 (s, 1H), 5.39 (d, *J* = 1.3 Hz, 1H), 4.72 (d, *J* = 12.4 Hz, 1H), 4.62 (d, *J* = 12.4 Hz, 1H), 4.39 (d, *J* = 1.3 Hz, 2H), 4.02 (dd, *J* = 4.8, 2.2 Hz, 1H), 3.97–3.88 (m, 4H), 3.83 (t, *J* = 9.4 Hz, 1H), 3.70–3.62 (m, 2H), 3.53 (dd, *J* = 11.8, 2.0 Hz, 1H), 1.26 (dd, *J* = 12.7, 6.1 Hz, 3H); ^13^C-NMR (101 MHz, CDCl_3_) δ 137.4, 134.9, 134.1, 133.0, 132.9, 131.3, 128.9, 128.5, 128.2, 128.1, 127.9, 127.8, 127.6, 127.3, 126.9, 126.1, 126.0, 125.9, 109.5, 86.6, 85.6, 79.6, 79.3, 77.8, 76.8, 75.7, 72.0, 71.8, 68.6, 61.5, 17.9.

**Phenyl 2,3,5-tri-*O*-benzoyl-α-l-arabinofuranosyl-(1→5)-** **α-l-arabinofuranosyl-(1→4)-3-*O*-benzyl-2-*O*-(2-naphthylmethyl)-1-thio-α-l-rhamnopyranoside 23**

Prepared from **11** and **22** according to the General Procedure I, but at −78 °C. White amorphous solid, 68%. R*_f_* 0.22 (toluene/EtOAc 4:1). ^1^H-NMR (300 MHz, CDCl_3_) δ 8.11–8.04 (m, 2H), 7.98–7.85 (m, 4H), 7.74–7.59 (m, 4H), 7.49–7.04 (m, 22H), 5.53 (d, *J* = 4.9 Hz, 1H), 5.50 (s, 1H), 5.42 (bs, 1H), 5.28 (s, 1H), 4.80–4.73 (m, 1H), 4.73 (s, 1H), 4.65 (d, *J* = 13.0 Hz, 1H), 4.60 (dd, *J* = 12.3, 5.1 Hz, 1H), 4.54–4.49 (m, 1H), 4.46 (d, *J* = 11.8 Hz, 1H), 4.38 (d, *J* = 11.8 Hz, 1H), 4.21 (d, *J* = 2.5 Hz, 1H), 4.03–3.91 (m, 6H), 3.87 (t, *J* = 9.3 Hz, 1H), 3.73 (dd, *J* = 10.9, 2.9 Hz, 1H), 3.66 (dd, *J* = 9.2, 3.0 Hz, 1H), 1.27 (d, *J* = 6.0 Hz, 3H); ^13^C-NMR (75 MHz, CDCl_3_) δ 166.1, 165.8, 165.3, 137.5, 135.1, 134.2, 133.5, 133.0, 133.0, 132.9, 131.1, 130.1, 129.8, 129.6, 129.5, 128.9, 128.6, 128.6, 128.4, 128.4, 128.2, 128.2, 128.1, 128.0, 127.8, 127.8, 127.6, 127.2, 126.6, 126.0, 125.9, 109.7, 106.0, 85.7, 85.5, 81.8, 81.8, 79.6, 79.3, 78.1, 77.4, 77.3, 75.5, 72.1, 71.5, 68.5, 67.1, 63.4, 18.1.

**Phenyl 2,3,5-tri-*O*-benzyl-α-l-arabinofuranosyl-(1→5)-2,3-di-*O*-benzyl-α-l-arabinofuranosyl-(1→4)-3-*O*-benzyl-1-thio-α-l-rhamnopyranoside S3**

Prepared from **5** according to the General Procedure V. White amorphous solid, 73%. R*_f_* 0.21 (toluene/EtOAc 10:1). ^1^H-NMR (300 MHz, CDCl_3_) δ 7.39–7.34 (m, 2H), 7.26–7.12 (m, 31H), 7.07 (dd, *J* = 6.6, 3.0 Hz, 2H), 5.47 (s, 1H), 5.45 (d, *J* = 1.3 Hz, 1H), 5.06 (s, 1H), 4.59 (d, *J* = 11.5 Hz, 1H), 4.52 (d, *J* = 11.2 Hz, 1H), 4.48–4.24 (m, 10H), 4.20–4.07 (m, 4H), 4.01–3.92 (m, 3H), 3.86–3.73 (m, 4H), 3.62–3.43 (m, 3H), 2.31 (brs, 1H), 1.24 (d, *J* = 6.2 Hz, 3H); ^13^C-NMR (75 MHz, CDCl_3_) δ 138.0, 137.8, 137.8, 137.4, 137.4, 137.2, 134.0, 131.2, 128.9, 128.5, 128.3, 128.2, 128.2, 128.2, 127.9, 127.8, 127.7, 127.6, 127.5, 127.5, 127.4, 106.9, 106.3, 88.3, 88.0, 86.9, 83.4, 83.3, 81.0, 80.6, 80.3, 75.4, 73.2, 72.1, 72.0, 71.8, 71.6, 71.3, 69.5, 69.3, 68.3, 66.1, 17.7. Copies of NMR spectra in Supplementary Materials.

**Phenyl 2,3,5-tri-*O*-benzyl-α-l-arabinofuranosyl-(1→5)-2,3-di-*O*-benzyl-α-l-arabinofuranosyl-(1→4)-3-*O*-benzyl-2-*O*-chloroacetyl-1-thio-α-l-rhamnopyranoside 24**

Prepared from **S3** according to the General Procedure VI. White amorphous solid, 94%. R*_f_* 0.52 (toluene/EtOAc 10:1). ^1^H-NMR (300 MHz, CDCl_3_) δ 7.41–7.33 (m, 2H), 7.28–7.09 (m, 31H), 7.07–7.01 (m, 2H), 5.59–5.55 (m, 1H), 5.47 (s, 1H), 5.34 (d, *J* = 1.5 Hz, 1H), 5.07 (s, 1H), 4.62 (d, *J* = 11.2 Hz, 1H), 4.50–4.38 (m, 7H), 4.38–4.20 (m, 4H), 4.20–4.09 (m, 3H), 3.99 (s, 2H), 3.97–3.93 (m, 3H), 3.88–3.80 (m, 3H), 3.75 (t, *J* = 10.8 Hz, 1H), 3.64–3.44 (m, 3H), 1.27 (d, *J* = 6.2 Hz, 3H); ^13^C-NMR (75 MHz, CDCl_3_) δ 166.7, 137.9, 137.7, 137.4, 137.3, 137.0, 133.4, 131.8, 129.0, 128.4, 128.3, 128.2, 128.2, 127.7, 127.6, 127.6, 127.5, 127.5, 106.9, 106.2, 88.1, 87.9, 85.7, 83.4, 83.3, 81.1, 80.6, 78.2, 75.4, 73.2, 72.1, 72.0, 71.7, 71.5, 71.3, 69.5, 68.7, 66.0, 40.7, 17.7.

**Pent-4-enyl 2,3,5-tri-*O*-benzyl-α-l-arabinofuranosyl-(1→5)-2,3-di-*O*-benzyl-α-l-arabinofuranosyl-(1→4)-3-*O*-benzyl-2-*O*-chloroacetyl-α-l-rhamnopyranosyl-(1→4)-6*-O-*acetyl-2,3-di*-O-*benzyl-β-d-galactopyranoside 2**

Furnished from **24** and **3** with general procedure VII. Colorless oil, 42%. R*_f_* 0.15 (toluene/EtOAc 10:1). ^1^H-NMR (400 MHz, CDCl_3_) δ 7.33–7.08 (m, 40H), 5.78 (ddt, *J* = 16.9, 10.2, 6.6 Hz, 1H), 5.55–5.52 (m, 1H), 5.39 (d, *J* = 7.2 Hz, 1H), 5.11 (d, *J* = 1.6 Hz, 1H), 5.07 (s, 1H), 5.02–4.89 (m, 2H), 4.87 (d, *J* = 11.1 Hz, 1H), 4.78–4.70 (m, 2H), 4.62 (t, *J* = 10.6 Hz, 1H), 4.50–4.39 (m, 6H), 4.39–4.21 (m, 8H), 4.16–4.04 (m, 4H), 3.98–3.81 (m, 8H), 3.82–3.68 (m, 5H), 3.66–3.41 (m, 5H), 2.17–2.08 (m, 2H), 1.98 (s, 3H), 1.79–1.68 (m, 2H), 1.23 (d, *J* = 6.1 Hz, 3H). ^13^C-NMR (75 MHz, CDCl_3_) δ 170.4, 166.2, 138.1, 137.9, 137.8, 137.8, 137.7, 137.7, 137.4, 137.4, 128.4, 128.3, 128.3, 128.2, 128.1, 127.9, 127.7, 127.7, 127.6, 127.5, 114.9, 106.7, 106.2, 103.9, 99.0, 88.2, 87.9, 83.3, 83.3, 81.0, 80.6, 80.5, 78.5, 77.8, 74.9, 73.5, 73.3, 73.2, 72.1, 72.0, 71.8, 71.6, 71.4, 71.2, 69.9, 69.7, 69.5, 68.3, 65.8, 62.5, 40.7, 30.1, 28.8, 20.7, 17.8.

## 4. Conclusions

The syntheses of two fully protected tetrasaccharides with diarabinan and digalactan side chains designed for assembly of larger RG-I oligosaccharides have been performed. The TMSOTf-promoted coupling of diarabinan and digalactan donors with a rhamnose thioglycoside acceptor gave two trisaccharide donors. These could be coupled selectively with an *n*-pentenyl galactoside acceptor. We envision that the prepared tetrasaccharides could be versatile building blocks for the synthesis of larger branched fragments of RG-I. We are currently working on the preparation of branched RG-I and examining if **1** and **2** can serve as glycosyl donors for this purpose.

## Supplementary Materials

Copies of NMR spectra for title compounds and **S1**–**S3** can be found in the Supplementary Materials.
